# Eliminating Race From eGFR Calculations: Impact on Living Donor Programs

**DOI:** 10.3389/ti.2022.10787

**Published:** 2022-11-11

**Authors:** Maria Irene Bellini, Mikhail Nozdrin, Maarten Naesens, Paulo N. Martins

**Affiliations:** ^1^ Department of Surgical Sciences, Sapienza University, Rome, Italy; ^2^ Faculty of Medicine, Imperial College London, London, United Kingdom; ^3^ Department of Microbiology, Immunology and Transplantation, KU Leuven, Leuven, Belgium; ^4^ Transplant Division, Department of Surgery, University of Massachusetts, Worcester, MA, United States

**Keywords:** kidney transplantation, end stage renal disease, race, ethnicity, living kidney donation, transplant access, inequity

The recent decision to remove race-based calculations of kidney function for candidates on the national waitlist approved by the OPTN Board has set the tone towards a more equitable assessment of prospective transplant and donor candidates (1). The change will take effect by the 27th of July in the USA and will allow hospitals to use only race neutral equations (without the black race coefficient) (2). This policy change alone will not likely address all the existing disparities in kidney transplantation (3, 4), but a reappraisal of the elimination of race from eGFR calculations is needed in view of its potential impact on living donor kidney transplantation (LDKT), the best treatment option for patients affected by end stage renal disease (ESRD), both from the donor’s and the recipient’s perspective.

In greater detail, there remains a disparity in providing equitable access to racial minorities (5), especially in areas where social-related status often limits access to care, as in the USA, where private insurance affects to the likelihood of treatment exposure and transplant referral: a recent analysis showed in fact that African American candidates have a lower incidence of LDKT than candidates of other races, regardless of primary payer (6). Furthermore, in Low- and Middle-Income countries, where deceased organ donation programs are not well-established, LDKT is the only curative treatment alternative to dialysis or death (7).

Evidence is lacking regarding ethnicity and organ donation in Europe. In fact, data collection is not generally undertaken and standardized, based mainly on self-identification or recorded country of birth. Furthermore, the discrepancy between national methodologies limits access to data for various minority groups, which in turn renders not only national, but also gathered European data collection less reliable than and less comparable to what happens in the USA (6). Additionally, in many countries, “race” data are simply not collected, primarily because it is felt that it could amount to racial discrimination; the flipside is that since the data are not there, it is not possible to fully assess the extent of racial discrimination in many ways.

In the UK, non-white ethnic minorities, comprise 11% of the population, 7% of organ donors, 35% of people awaiting a kidney transplant and 21% of people who died on the waiting list (7). In other European countries, the situation is similar to or worse than that described in the UK, and in Norway, one of the countries with the highest LDKT rates, living organ donation appears to be rare amongst migrant and ethnic minority groups, who then rely upon organs from deceased donors (8), with mitigation for the disparity in access to kidney care between ethnic groups being advocated worldwide (9).

Demographic characteristics of donors (10), recipients (11), and the interaction between these two (12), are increasingly considered in the establishment of research protocols and healthcare policies. To achieve better outcomes, and in consideration of the known discrepancy in life expectancy and morbidity between different ethnicities, it is therefore of utmost importance to consider comprehensively the interrelation between donor and recipient races on the respective health outcomes, to provide equitable access to individuals of different socio-racial backgrounds, yet without a further exacerbation of the already existing inequalities.

Race is a variable often considered in eGFR calculations, with the potentiality to overestimate renal function in Black patients, causing about 16% misclassification of kidney disease stage (2), and thus exacerbating health inequalities by the miscalculation of kidney function in minority groups. The equations most in use today include serum creatinine, age, sex, and race, and adjust the final calculation based on a presumed higher muscle mass in Black individuals; this applies specifically for the commonest methods in use among adults, namely the Modification of Diet in Renal Disease (MDRD) and the Chronic Kidney Disease Epidemiology Collaboration (CKD-EPI) (13). Yet, there are additional social determinants of health in relation to income, education and general lifestyle conditions that could significantly affect the final eGFR calculation. Furthermore, most of the eGFR equations were originally developed considering a relatively small sample size and with limited demographic characteristics (i.e., White men), therefore their transferability to other backgrounds could be argued in view of the lack of inclusion of other specific demographic characteristics for the calculation of the equation itself and for its original validation, in contradiction with the principles of diversity and inclusion.

As a result of health inequality, Black and Asian minority people in need of a kidney transplant wait for longer in comparison to their Caucasian counterparts (14). This has also been proven for Hispanic ethnicity and female gender (15), where lack of formal education and minority race are negatively associated with referral to a transplant center (14). The extended time on the waiting-list unfortunately often leads to a deterioration of the general health conditions to a grade at which the underlying comorbidities of these candidates cause their ineligibility to undergo kidney transplantation, mostly because of the limited organ donor pool, with the sad result of death for many.

To possibly meet the organ donor offer, LDKT not only represents the best opportunity of success in terms of definitive renal replacement therapy, but it also allows pre-emptive treatment of kidney failure. Since LDKT is unfortunately a precious resource not available for everyone, educational campaigns aiming to expand living organ donation should target these minority backgrounds, and content related to risks for the altruistic act of donation by Black and Asian candidates should cover topics related to the effects of donor and recipient races on the respective health outcomes.

What is then the available evidence on the effect of race on living kidney donors, and the impact on recipients’ outcomes? As previously stated, data on post-donation eGFR might be affected by the formulas used in the calculation, so they remain heterogenous and inconclusive, therefore a more accurate analysis could focus on the percentage change in eGFR or slope eGFR in longitudinal observations (11) or in a comprehensive assessment evaluating biological data, socioeconomic status, and eventual complementary data affecting the health-related status of an individual.

In greater detail, we previously demonstrated that race, *per se*, should not be a barrier to increase the living donor kidney pool: on average, 88% of the entire living donor pool of this international cohort are Caucasian, but with the help of the previous mentioned educational campaigns, up to 40% of Black and Asian minorities have proven to be a realistic target to contribute to the living organ donor pool (16).

If we look at the incidence of proteinuria, another important parameter to assess the parenchymal damage secondary to the compensation hyperfiltration of the remnant kidney, there seems to be no difference among Africans or Caucasians (8) 1-year post-donation, thus confirming that living donation is an option for all the races to increase chances and access to transplantation.

Besides, there is no difference in incidence of ESRD between the Caucasian and Asian or Hispanic/Latin ethnic backgrounds (8), thus providing further support to the hypothesis that in addition to just genetic conditions, there are factors such as socioeconomic deprivation and racial discrimination to be considered for the long-term outcomes.

To this regard, an analysis from the OPTN/UNOS database found significantly higher rates of ESRD in African donors compared to Caucasians: Lentine et al., adjusted HR 2.32 (1.48–3.62) *p* < 0.001 (17). There has also been higher incidence of ESRD reported in both Caucasian and African donors, in comparison to their healthy counterparts in the general population (10); however, more than three times higher ESRD rates in the general population are registered in African adults, 8%, compared to Caucasians, 2%–3% respectively, leading ultimately to a further disadvantage of African donors and creating a vicious cycle. Therefore, it is compelling to protect those who come forward for a generous act of self-giving, without additional harm secondary to a racial demographic.

Finally, if we look at what happens to Black kidney transplant recipients, in a recent meta-analysis we demonstrated no significant difference between the 1-year mortality in comparison to Caucasians (11), as well as with regards to the data on acute rejection, concluding that recipient’s race is not related to patient and graft survivals (11).

In conclusion, Black deceased donors are more likely to experience CKD compared to Caucasians, mainly in view of the trends present in the general population.

This should not be considered a barrier to the expansion of the living donor pool and the possibility to offer LDKT to candidates of Black and Asian minorities should instead be concrete and actively incentivized.

The new proposed OPTN/UNOS race-neutral eGFR calculations (13) might be considered sufficiently accurate for clinical practice in many circumstances but may lead to systematic differences in accuracy of eGFR between race groups, with implications for individual patients and public health. There have also been some concerns that the elimination of the black coefficient would decrease the eGFR and reduce the eligibility of potential black living donors, although this concern is not valid because most if not all centers do not use eGFR in the workup for living donors (4), but more reliable tests or 24 h urine clearance.

We believe that future studies need to focus on how to overcome this barrier in consideration of the current organ donor shortage, to minimize the effect of race in kidney function and provide equitable access to individuals of different socio-racial backgrounds. We also strongly support the omission of adjustment for ethnicity in the eGFR formulas, in agreement with current research looking at new endogenous filtration markers and interventions to eliminate racial and ethnic disparities, supporting consideration in health outcome differences due to health inequalities rather than race.

Transplant and Nephrology Societies should favor this new policy change to intervene on the long overdue negative impact of race on eGFR, with the aim to reduce delayed referrals for transplant and delays in qualifying for waiting time and for donor’s eligibility. Equity in health means “equal opportunity” (18) and thus patients should all start from equal assessment to be offered equal treatment options.

## Data Availability

The original contributions presented in the study are included in the article/Supplementary Material, further inquiries can be directed to the corresponding author.

## Abbreviations

CKD, chronic kidney disease; eGFR, estimated glomerular filtration rates; LDKT, living donor kidney transplantation; ESRD, end stage renal disease.
